# Recurrence plot embeddings as short segment nonlinear features for multimodal speaker identification using air, bone and throat microphones

**DOI:** 10.1038/s41598-024-62406-3

**Published:** 2024-05-31

**Authors:** K. Khadar Nawas, A. Shahina, Keshav Balachandar, P. Maadeshwaran, N. G. Devanathan, Navein Kumar, A. Nayeemulla Khan

**Affiliations:** 1 School of Computer Science and Engineering, Vellore Institute of Technology, Chennai, Tamilnadu 600127 India; 2 Department of Information Technology, Sri Sivasubramaniya Nadar College of Engineering, Kalavakkam, Tamil Nadu 603110 India

**Keywords:** Nonlinear dynamics, Recurrence plot (RP), Recurrence plot embeddings, Multimodal speaker identification, Bone microphone, Throat microphone, Convolutional neural networks (CNN), Computer science, Information technology

## Abstract

Speech is produced by a nonlinear, dynamical Vocal Tract (VT) system, and is transmitted through multiple (air, bone and skin conduction) modes, as captured by the air, bone and throat microphones respectively. Speaker specific characteristics that capture this nonlinearity are rarely used as stand-alone features for speaker modeling, and at best have been used in tandem with well known linear spectral features to produce tangible results. This paper proposes Recurrent Plot (RP) embeddings as stand-alone, non-linear speaker-discriminating features. Two datasets, the continuous multimodal TIMIT speech corpus and the consonant-vowel unimodal syllable dataset, are used in this study for conducting closed-set speaker identification experiments. Experiments with unimodal speaker recognition systems show that RP embeddings capture the nonlinear dynamics of the VT system which are unique to every speaker, in all the modes of speech. The Air (A), Bone (B) and Throat (T) microphone systems, trained purely on RP embeddings perform with an accuracy of 95.81%, 98.18% and 99.74%, respectively. Experiments using the joint feature space of combined RP embeddings for bimodal (A–T, A–B, B–T) and trimodal (A–B–T) systems show that the best trimodal system (99.84% accuracy) performs on par with trimodal systems using spectrogram (99.45%) and MFCC (99.98%). The 98.84% performance of the B–T bimodal system shows the efficacy of a speaker recognition system based entirely on alternate (bone and throat) speech, in the absence of the standard (air) speech. The results underscore the significance of the RP embedding, as a nonlinear feature representation of the dynamical VT system that can act independently for speaker recognition. It is envisaged that speech recognition too will benefit from this nonlinear feature.

## Introduction

Speaker recognition has broad applications that include authentication (voice biometrics), surveillance (electronic eavesdropping of telephonic conversation), forensics (spoof detection in criminal cases), multi-speaker tracking, and personalized user interface, among others. The identity of a speaker depends, to a large extent, on the physiological aspects of the vocal tract mechanism, a nonlinear dynamical system that produces speech. Speech, which is a uni-dimensional measurement of this dynamical system, is transmitted through multiple modes- air, bone, and skin conduction. Each of these signals contains information about the nonlinear dynamics of the vocal tract system that are speaker-specific. Also, in certain situations, the amount of speech signals available to evaluate a speaker recognition system could be far less than required. In real time applications, problems related to memory and computational resource limitations necessitate recognition from short speech utterances^1^. In applications that involve forensics or access control, it is less likely to get sufficient data even for enrollment (training)^2^. This paper attempts to address these issues by proposing a stand-alone, short segment feature that independently (without being used in combination with well-proven linear features) and effectively captures the speaker-specific nonlinear dynamics of the vocal tract system in all three acoustic modes of speech for building a multimodal speaker recognition system. This system gives a high accuracy of 99.84%. The contributions of the paper are:

1. It proposes recurrent plot embedding as a new nonlinear dynamical feature that captures the speaker information. Unlike the earlier works, it acts as a stand-alone feature (without using any linear feature in tandem with it) to give a multimodal speaker recognition performance on par with state-of-the-art systems.
2. It demonstrates that speaker-specific nonlinear dynamics underlying the speech transmitted through bone and throat skin conduction could be effectively captured using recurrent plot embeddings. This joint feature space could also be used to build effective bimodal speaker recognition systems using alternate speech sensor data alone. Such a system would be useful in situations where air conduction speech data cannot be used or is not available.
3. It reduces the duration of a test utterance to 37.5 ms and still gives a performance that is comparable with state-of-the-art systems. This duration is shorter than all other earlier works on short segments for speaker modeling.

## Nonlinearity, multiple modes, and short utterances for speaker modeling.

This section gives a brief overview of features used for speaker recognition in “Features for speaker recognition” section. It then discusses the three aspects of speaker modeling that this paper addresses, namely nonlinear dynamics of the vocal tract system in the section “Nonlinear dynamics of vocal tract for speaker modeling”, multimodal systems in section “Multimodal systems for speaker modeling”, and short segments of speech in section “Short speech segments for speaker modeling”, along with a review of the works of earlier and contemporary researchers in each of these aspects.

### Features for speaker recognition

Commonly, for speaker verification tasks, features such as Mel Frequency Cepstral Coefficients (MFCCs), Perceptual Linear Prediction (PLP) coefficients, and Linear Prediction Cepstral Coefficients (LPCCs) based on short term spectral analysis are employed. In a systematic review^3^, the authors discuss the numerous variants of MFCCs, PLP, LPCCs and their fusion with other features used for speaker recognition. They report MFCC variants are the predominantly popular base features. For short utterance ($<10$ s) speaker recognition task, variants of Gammatone Frequency Cepstral Coefficients (GFCCs) were shown to perform better than a baseline MFCC based system^4^.

These features however do not directly capture the non-linear dynamics of the speech signal, as they focus on extracting features related to the spectral envelop of the speech signal and inherently ignore the phase relationship between the frequency components^5^. Focusing on the piecewise linearity of the speech signal to extract features ignores crucial information on the non-linear dynamics, making the information being extracted incomplete^6^.

Deep embeddings that are obtained by converting the time-frequency domain speech utterance into a high-dimensional feature vector/embedding for speaker recognition are commonly employed in recent research. In the identity vector (i-vector) approach^7^, a low-dimensional representation of speaker and channel variations is created using simple factor analysis. Deep vectors (d-vector) are created by training a deep network to classify speakers at the frame level using context stacked acoustic features^8^. The last layer of the trained DNN gives speaker specific features which are averaged for an utterance to form the d-vector. In an x-vector system, a DNN tuned to discriminate between speakers maps utterances of variable lengths into a fixed dimensional embedding^9^. The x-vector’s, fixed-dimensional embeddings was shown to outperform the traditional i-vector system for short utterances^10^. A short survey of various speech embeddings used as front-end for speaker recognition is reported in^11^. In this study we use similar deep embeddings generated based on recurrence plots.

### Nonlinear dynamics of vocal tract for speaker modeling

Traditionally, speakers are modeled using the linear, time-varying source-filter model of speech. The features used include well-known features such as Mel Frequency Cepstral Coefficients (MFCC), Linear Prediction Cepstral Coefficients (LPCC), etc. However, the speech production system exhibits nonlinear dynamics^12–14^. Teager and Teager^15^ in their classic paper have shown through experiments that, during speech production, the airflow within the vocal tract comprises jets tangential to and along the wall of the cavity and large radial and axial vortexes. The jets of airflow have a variety of interactions among themselves, leading to the conclusion that the vocal tract exhibits an oscillatory, nonlinear, aerodynamic phenomenon during speech production, contrary to being a passive acoustic system. The resulting complex behavior of the speech produced by this nonlinear deterministic system is considered chaotic and thus cannot be modeled using linear differential equations.

Takens theorem^16^ is used in^12^ to analyze the nonlinear dynamics of the speech signal by estimating the three dynamical attributes, namely Lyapunov exponents, metric entropy, and Correlation Dimension (CD). These attributes were estimated by reconstructing the state-space trajectories of the underlying vocal tract system using two optimality criteria methods, namely, singular value decomposition and redundancy. Tao et al.^17^ used a chaos detection technique called chaotic titration using the Volterra–Wiener nonlinear identification method to show that the chaotic component in speech is present in its LPC residual error. This interesting observation could help in modeling the excitation source as a chaotic source (instead of using a series of linear pulses as excitation) to improve the quality of speech coding or speech synthesis, etc. Alternatively, Fractal dimensions (FD) that were estimated from the multidimensional phase space reconstructed from the speech signal were used in^18^ to characterize speech sounds. FD exhibited a correlation with MFCC, which was higher for vowels than for fricatives and stop sounds. Dimitriadis et al.^19^ explored an amplitude modulation–frequency modulation (AM–FM) approach to model some nonlinear mechanisms of speech production^12^.

The physiological and behavioral characteristics of the speech production system determine the identity of the speaker. This speech production system is a nonlinear, deterministic one, so a suitable parametric representation of a speaker that is derived from the nonlinear deterministic dynamics and that which has minimum variability for a speaker and maximum variability for different speakers is necessary. Bandt and Pompe^20^ did a preliminary study on the use of entropy profiles as a nonlinear feature representation of the speech signal to distinguish speakers. Petry and Barone ^21,22^ demonstrated an improvement of 2–3% in the performance of the speaker recognition systems when nonlinear features were used in tandem with linear features. Their results showed the presence of speaker information in the nonlinear features that were in addition to (and not present in) the speaker context in linear features. At the same time, the nonlinear feature used in the former was fractal dimension (added to LPCC), and the latter used Kolmogorov (or metric) Entropy (KE) and largest Lyapunov exponent (LLE) in addition to FD. Kumar et al.^23^ combined six nonlinear features, namely KE, LLE, CD, capacity dimension, Eigenvalues of reconstructed phase space, and spectral delay coefficients, to improve the performance of the speaker recognition system. In^21^ among all the studies, the best performance of 97.3% was achieved only when nonlinear dynamical features were combined with linear features. They showed that adding nonlinear dynamic features to standard features like LPCC is equivalent to adding speaker dependent features not present in the standard features. It was achieved with short duration (1.2 s) training data.

However, these nonlinear features cannot be used as a stand-alone feature to achieve a performance that is anywhere close to what state-of-the-art techniques (using the linear feature) achieve. These recent techniques are based on deep learning architectures such as Convolutional Neural Networks (CNN)^24^, Wav2Vec2.0^25^, Deeper Feature CNN-Connectionist Temporal Classification (DFCNN-CTC)^26^, Gated Recurrence Unit-CNN (GRU-CNN)^27^ etc.

### Multimodal systems for speaker modeling

In order to increase the performance of speaker recognition in adverse conditions such as noise, multimodality has been explored by numerous researchers. Alternate supplementary information such as lip reading^28^, speech recorded with non-invasive sensors like throat microphone^29^ and bone conduction microphone^30^ have been shown to provide large gains in performance in adverse noisy conditions. Information about the speaker is present in all audio modes of speech, whether conducted through air, bone, or skin. Alternate (bone and throat) speech signals are relatively robust to the ambient noise compared to the standard (air conduction) speech. Hence researchers have integrated several alternate speech sensors along with the standard speech to build multimodal speaker recognition systems with improved performances over unimodal systems in nonstationary (multiple speakers with background), noisy environments^31,32^ combined the distinct signals from Glottal Electromagnetic Micropower Sensor (GEMS), Electroglottograph (EGG), and Physiological Microphone (P-Mics) to build a multimodal speaker recognition system. Linear transformation features of normalized speech segments, reduced in dimension, were given as input to Gaussian Mixture Models and Support Vector Machine classifiers. A late integration with standard microphone signals resulted in improved performance of 95.8% accuracy. Other researchers such as^33–36^ too have explored throat microphone, bone conduction microphone GEMS EGG, and non-audible murmur microphone signals’ combination with standard speech for improving the speaker modeling. Linear features such as LPCC, MFCC, and i-vectors were used in all these works. In all these studies, the nonlinearity associated with the skin and bone conduction was not considered.

### Short speech segments for speaker modeling

The duration of speech utterances for evaluating a speaker recognition system is a cause of concern when there is access only to a limited amount of speech data and when there is large intersession variability within a speaker. Kanagasundaram et al.^37–39^, Vogt et al.^40^, Kenny et al.^41^, McLaren et al.^42^ have focused on building reliable speaker recognition systems that use reduced duration of speech for evaluation. Some of the techniques used are Joint Factor Analysis (JFA)^43^, i-vectors^44^, support vector machines^44^ and Probabilistic Linear Discriminant Analysis (PLDA)^39,45^. In^46^ Gaussian PLDA and heavy-tailed PLDA were used to model speakers with limited (10–5 s) speech. In^44,47^, JFA and i-vector techniques were used on as little as 2 s of speech data for evaluating the speaker recognition system.

Some of the gaps identified in the review of the literature are:

- There is a challenge in achieving high performance in speaker recognition systems based on short segment speech because the shorter the speech segment, the greater is the intra-speaker variability^48,49^.
- Earlier works on multimodal speaker recognition systems have shown that performance improved either by using bone microphone speech or throat microphone speech in tandem with air microphone speech, as each of these alternate sensors capture complementary evidence. No work has combined both these complementary pieces of evidence, especially as an alternative to standard air microphone features, for situations (e.g., stealth operations) where the latter may not be available.
- Earlier works on nonlinear speaker specific features used them in combination with linear features to augment the performance of the speaker recognition system, and not as a stand alone feature.

This work, for the first time (to the best knowledge of the authors), introduces recurrent plot embeddings, a short duration and nonlinear dynamical feature that acts as a standalone feature and exclusively (without the support of linear features) helps identify speakers in a multimodal framework with an accuracy that is similar to state-of-the-art speaker recognition systems built using linear features.

The rest of the paper is organized as follows: Section “Characterization of Recurrent Pattern of Chaotic vocal tract system” builds a case for recurrent plot embeddings as a representation of speaker information. The experimental studies on speaker recognition are detailed in “Experimental studies on speaker recognition” section, while the “Experimental results” section analyses the experimental results. In “Discussion” section discusses the results and “Conclusion” section concludes with a highlight of the salient features of this work.

## Characterization of recurrent pattern of chaotic vocal tract system

A speaker produces speech with a nonlinear, oscillatory vocal tract system (VT)^15^. This speech is defined by the two nonlinear partial differential equations^50^:

1 $$\begin{aligned} k \frac{\partial p}{\partial t}= & {} - \frac{\partial u}{\partial x} - ku \frac{\partial p}{\partial x} \end{aligned}$$

2 $$\begin{aligned} \frac{\partial p}{\partial x}= & {} - \rho \left[ \frac{\partial u}{\partial t} + u \frac{\partial u}{\partial x}\right] \end{aligned}$$

where $\frac{\partial p}{\partial x}$ and $\frac{\partial u}{\partial x}$ are the differential pressure and differential flow across a section respectively and $u\frac{\partial u}{\partial x}$ is a convection term. Likewise $\frac{\partial p}{\partial t}$ denotes the rate of change of acoustic pressure, while $\frac{\partial u}{\partial t}$ denotes the rate of change of flow velocity. *k* is the inverse of the bulk modulus, and $\rho$ the density. The unique flow patterns of various sounds are a combination of separation flows, axial vortexes, radial vortexes and interaction among them. These sounds capture the nonlinear dynamics of the VT system. This VT system is unique to each speaker. In order to understand the underlying nonlinear dynamics of the multidimensional, oscillatory VT system, from the observable 1D speech signal, a suitable feature must be extracted. Spectrograms and Fourier analysis, though they give information on the spectral content of the VT system, fail to capture its nonlinear dynamics.

### Reconstructed Phase space

The analysis of the dynamics of the low-dimensional, chaotic VT system, *v*(*t*), could be done by reconstructing (embedding) its full motion in the *n*-dimensional phase space, from the speech signal, *s*(*t*) (which is of a single degree of freedom). This motion involves the evolution in time *t*, of the vector, *v*(*t*), constructed out of time-delayed copies of *s*(*t*) given by ${v(t)} = [{s(t)}, {s(t + \tau )}, {s(t + 2\tau )}, \ldots , {s(t + n\tau )}]$, where $\tau$ is the delay time, and *n* is the embedding dimension, ${v} \in \mathbb {R}^{n}$ and ${s} \in \mathbb {R}$. The trajectory of *v*(*t*) in the *n*-dimensional reconstructed phase space, ($\mathbb {R}^{n}$: ${v(t_{0})}$ $\rightarrow$ *v*(*t*)), is related to the trajectory of the original vocal tract system in the full phase space ($\mathbb {R}^{d}:$ ${x(t_{0})}$ $\rightarrow$ *x*(*t*)). Thus from the dynamics of the one dimensional speech signal, we can recreate the dynamics of the *n*-dimensional VT system. This embedding is possible because though the other degrees of freedom of the VT system are hidden, they affect the dynamics (time evolution) of the speech signal. From the Reconstructed Phase Space (RPS) characterizations, the nonlinear dynamics of the VT system such as fractal dimension, Lyapunov exponents, entropy, among others have been estimated^51–54^.

### Recurrence plot embeddings

Consider a one-dimensional, windowed speech signal of size *N*, given by:

3 $$\begin{aligned} S = {s_{1}, s_{2}, \ldots s_{N}}, s \in \mathbb {R} \end{aligned}$$

The dynamics of the VT system can be reconstructed in phase space, where the state of the VT system at a given instant in its trajectory is given by:

4 $$\begin{aligned} \varvec{v_{i}} = \left[ \varvec{s_{i}}, \varvec{s_{i + \tau }}, \varvec{s_{i + 2\tau }},\ldots , \varvec{s_{i + (n-1)\tau }}\right] , \varvec{v_{i}} \in \mathbb {R}^{n} \end{aligned}$$

where *n* is the embedding dimension, and $\tau$ is the delay parameter.

Recurrent patterns in *S* reflect recurrent patterns in the dynamics of the oscillatory VT system. It is known that this oscillatory VT system has an anatomical structure that differs among members of the same gender, as well as between the genders^55–57^. This structure contributes to both inter- and intra-speaker variability in speech. For example, the (variations in the shape, mass and elasticity of the) vocal cords are responsible for both inter- and intra-speaker variability in speech. Additionally, the vocal tract between the glottis and the lips, as captured by parameters such as ratio of the length of the pharyngeal portion to the oral portion, configuration of the teeth and palate, size and configuration of the nasal cavity differ appreciably among individual speakers, and contribute primarily to the inter-speaker variability^58^. The complex interactions between the vocal cords, tongue, lips and other articulators results in specific shapes and configurations of the vocal tract at any moment during articulation of speech^58^. This latent dynamics of the vocal tract is effectively captured in the geometric patterns of the Recurrent Plots (RPs). These recurrent patterns capturing the dynamics of the oscillatory vocal tract system could then be said to be unique to each of the VT system and hence unique to each speaker. Since the geometric patterns in the RPs capture the latent, dynamic behavior of the vocal tract system and source characteristics, they could be used to compare the behavior of different vocal tracts (here, speakers). This is because speaker specific information is present in both system and source characteristics^59^. In other words, the RPs could be used for the classification of speakers. An RP of a speech signal of size *N* is a matrix of distances between all the $N^2$ possible vectors, and is given by:

5 $$\begin{aligned} \varvec{T_{ij}}= & {} \Theta (\epsilon - ||\varvec{v_{i}} - \varvec{v_{j}}||); \quad i,j = 1, 2\ldots N \end{aligned}$$

6 $$\begin{aligned} \varvec{\Theta }= & {} {\left\{ \begin{array}{ll} \textbf{1} &{} \epsilon > ||. || \text { (represented by black pixel)}\\ \textbf{0} &{} \text {otherwise} \text { (represented by white pixel)} \end{array}\right. } \end{aligned}$$

where $\varvec{v_{i}}$ and $\varvec{v_{j}} \in \mathbb {R}^{n}$ are points in Reconstructed phase space, $\epsilon$ is the threshold, $\Theta$ is the Heaviside function and $||\varvec{v_{i}} - \varvec{v_{j}}|| = \max _{k} ({\varvec{s_{i+k}} - \varvec{s_{j+k}}}), k = 1, 2, \ldots n$. i.e., the maximum distance along any $k^{\text {th}}$ dimension is chosen as the distance between the two vectors. A historical review and the application of recurrence based methods is seen in^60,61^.

**Figure 1:**
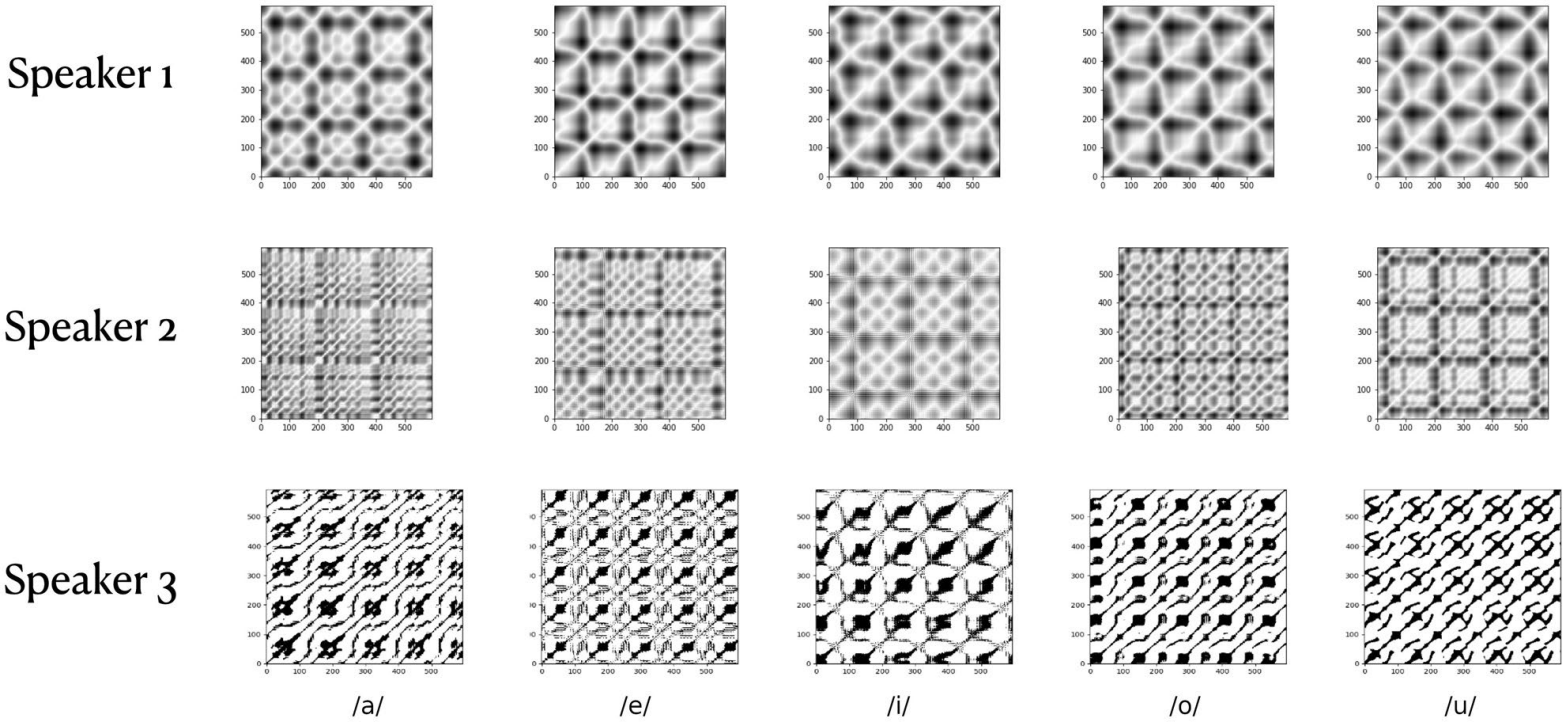
The figure shows the recurrence plots of the vowel segments of the sounds; /ba/, /be/, /bi/, /bo/, /bu/, spoken by 3 different speakers.

**Figure 2:**
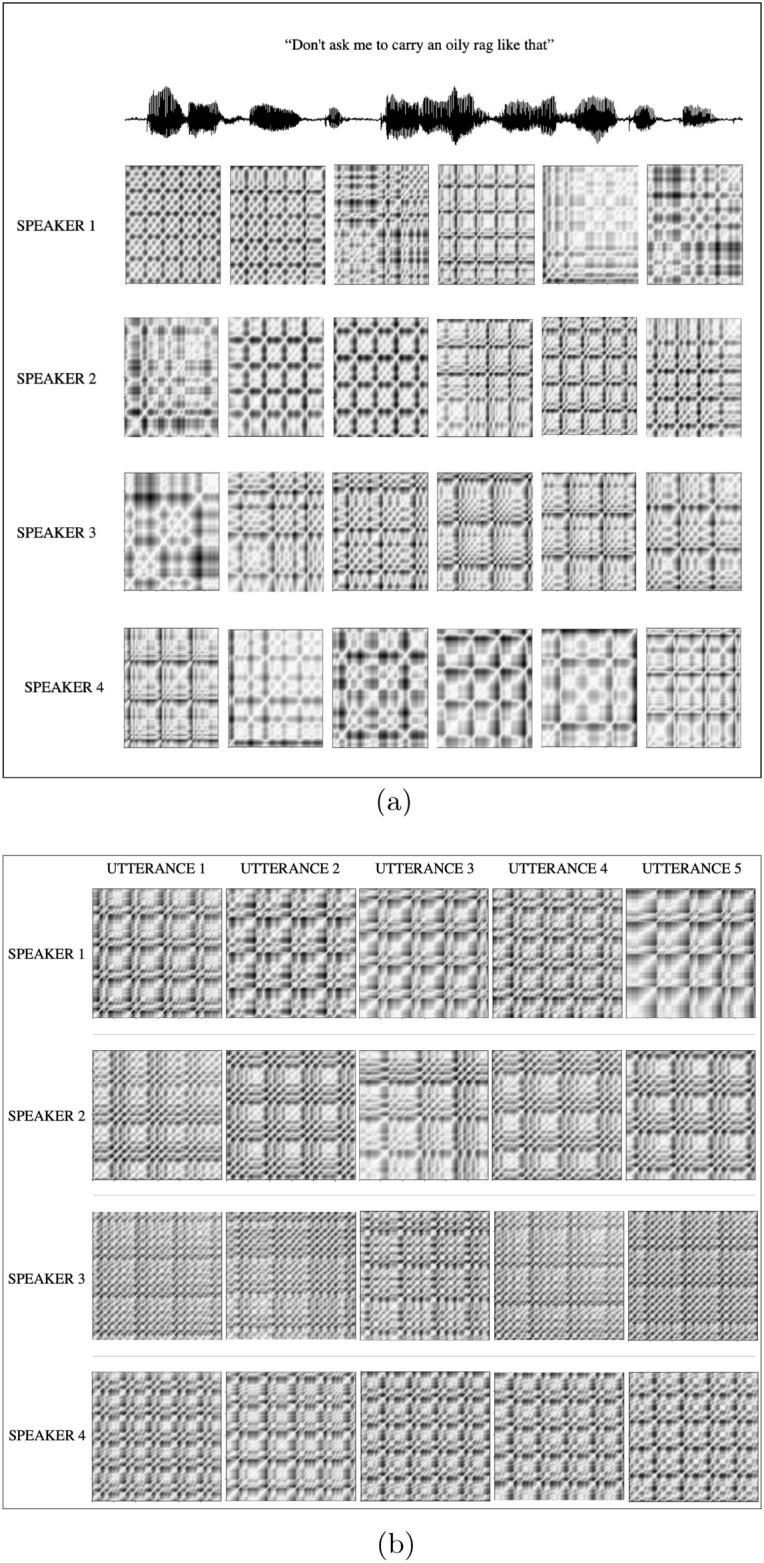
Inter and Intra-speaker variability.

To generate the recurrence plots in this study, the pyts toolbox is used with an embedding dimension = 2 and time delay = 6, determined based on the false nearest neighbor method and average mutual information, respectively. Figure 1 shows the RP for different sound units (specifically five different vowels used in Indian languages, /a/, /e/, /i/, /o/ and /u/) for different speakers, and Fig. 2a shows the consecutive RP derived from continuous speech, for different speakers. Figure 2b shows different instances of the RPs for the same sound unit /bha/ for each speaker. In Fig. 1 a comparative observation of the first column of RPs corresponding to vowel /a/ of the three speakers shows that RPs are different for each speaker. So is the case for each of the 5 vowels. Similarly, in Fig. 2a, a visual observation (comparing the row sequences) shows that no two sequences of RPs are similar. These two figures illustrate the inter-speaker variability. In contrast, Fig. 2b shows RPs for five different utterances of the same speaker for the syllable /bha/, in each row. We observe that though there are variations between speakers, there are subtle variations in the RPs within utterances of the same speaker. This shows that RPs capture the intra-class variability as well.

Since the RPs capture the dynamics of the vocal tract system more than that of the vocal cords, they naturally have more inter-speaker variability and less intra-speaker variability.

The matrix $\varvec{T}$ (RP Portrait) is the input to the convolutional neural network (CNN). The output of the $1^{\text {st}}$ Convolutional layer is given by:

7 $$\begin{aligned} A^{1}_{ij} = g^{1} \left( \varvec{\sum _{m} \sum _{n} W_{m,n}^{1}} \varvec{T_{i+m, j+n}} + b^{1}\right) \end{aligned}$$

While the output of the final convolutional layer *L*, the feature map is given by:

8 $$\begin{aligned} A^{L}_{ij} = g^{L} \left( \varvec{\sum _{m} \sum _{n} W_{m,n}^{L}} \varvec{A_{i+m, j+n}^{L-1}} + b^{L}\right) \end{aligned}$$

where $W_{m,n}^{L}$ is the weight matrix (kernel of dimension $k \times k$) connecting any two consecutive layers *L* and $L-1$, $b^{L}$ is the bias of the layer *L* and $g^{L}$ is the ReLU activation function.

### Feature maps

The feature map $\varvec{A^{L}}$ corresponding to the input recurrence plot *T* thus obtained are class-discriminative, as visualized using the Gradient-weighted Class Activation Mapping (Grad-CAM)^62^. The Grad-CAM produces coarse heat maps, *H*, which is a linear weighted combination of $A^{L}$.

9 $$\begin{aligned} H = \text {ReLU} \left( \sum _{L} \alpha ^{C}\varvec{A^{L}}\right) \end{aligned}$$

where the weight $\alpha ^{C}$ captures the significance of $A^{L}$ for a target class *C*. in other words, it captures the features ($y^{C}$ ) or pixels that positively influence the score of class *C*. $\alpha ^{C}$ is obtained by the average-pooling of the gradient $\frac{\partial y^{C}}{\partial A^{L}}$ as:

10 $$\begin{aligned} \alpha ^{C}_{L} = \frac{1}{z}\sum _{i}\sum _{j} \frac{\partial y^{C}}{\partial A^{L}}_{i,j} \end{aligned}$$

**Figure 3:**
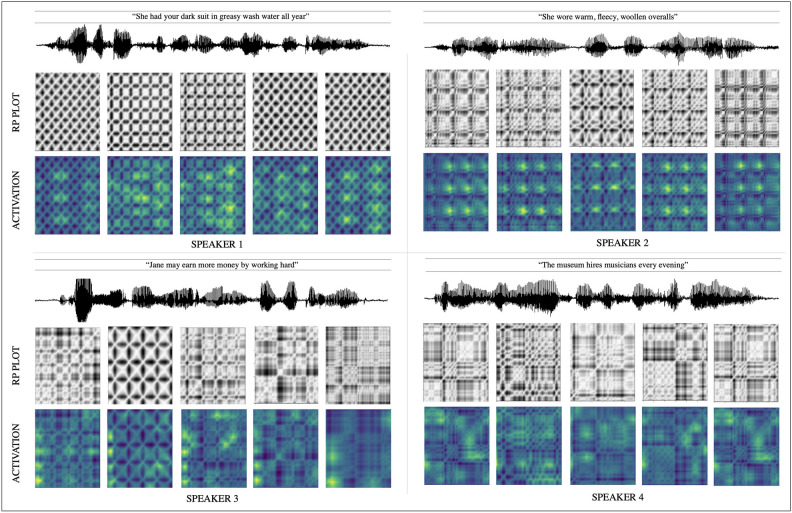
RP portraits and the corresponding class discriminating activation maps that were generated for five consecutive frames of size 600 samples for four different speakers.

The heat maps or class-discriminating activation maps corresponding to a sequence of recurrence plots obtained from two different speakers for the same utterance is shown in Fig. 3.

## Experimental studies on speaker recognition

**Figure 4:**
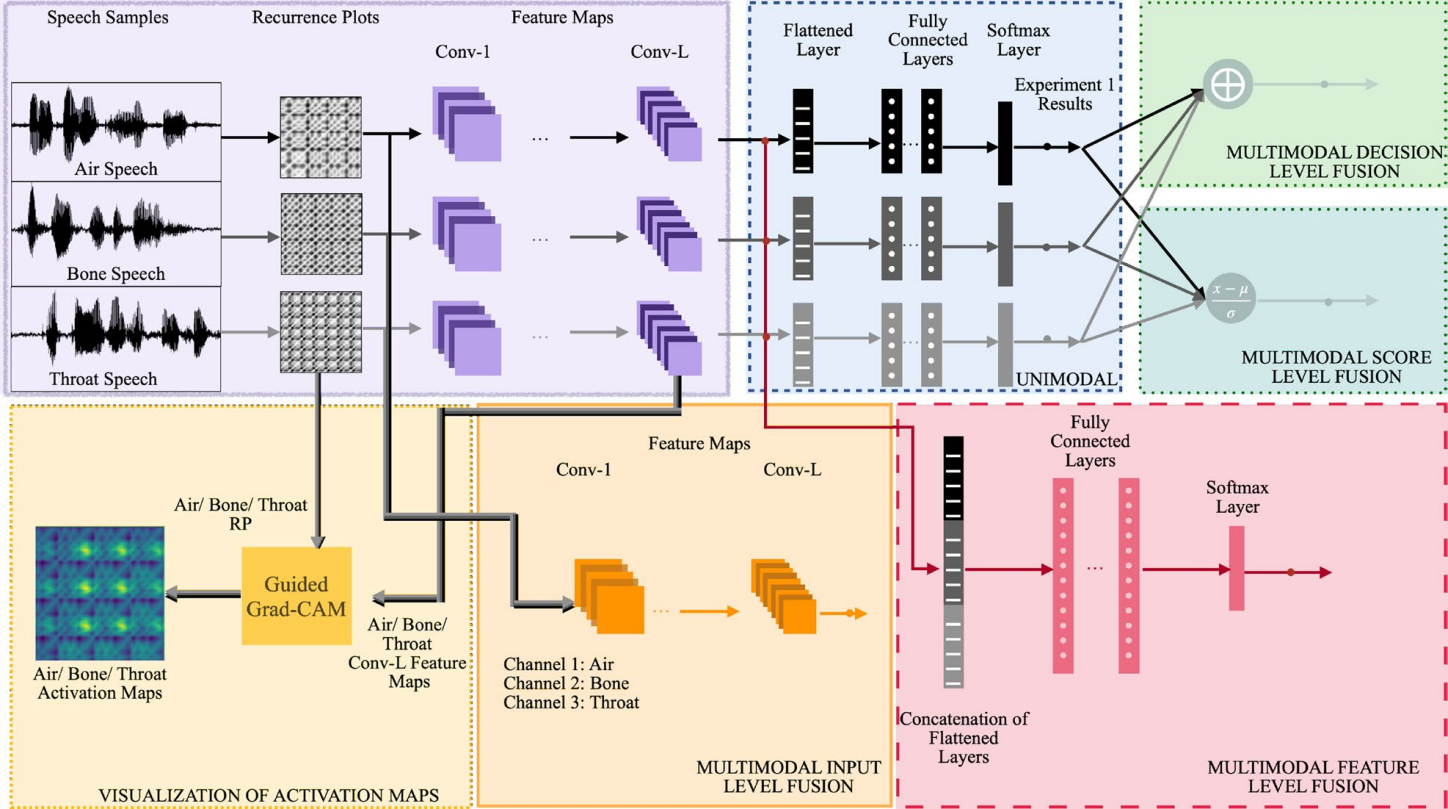
Block diagram depicting the unimodal, bimodal and trimodal speaker recognition systems used in this study.

This section discusses the experimental setup of the work including the datasets used, features, deep learning architectures used, training and testing, and performance evaluation of the various text independent speaker recognition systems. Figure 4 shows the block schematic of the setup.

### Datasets

In this study, we perform closed-set speaker identification experiments on two datasets. For evaluating the unimodal, bimodal and trimodal speaker recognition systems, we use:

1. Continuous (Multimodal) TIMIT speech corpus, referred to as the CMT corpus throughout this paper.
2. Consonant-Vowel (Unimodal) syllable dataset, referred to as CV dataset in this paper.

Each of these datasets has been designed/used in this work for evaluating both singular and joint RP embedding space for modeling the nonlinear speaker characteristics using unimodal and multimodal approaches. The speaker identification experiments on the CMT corpus are to demonstrate the speaker-discriminative ability of the joint RP embeddings of the multimodal data obtained from complementary modes (air, bone, and throat) and to evaluate the generalization ability of the CNN-based multimodal system in learning the join feature space. On the other hand, the vowel regions in the CV dataset are regions of sustained vocalization. These sustained vocalizations have better information about the nonlinear vocal tract system^63^. It would be interesting to study and compare how well various nonlinear speech characterizations such as the proposed (singular) RP embeddings and those features mentioned in the literature such as Lyapunov exponents, fractal dimension, entropy, etc. that capture the nonlinearity during sustained oscillations that are speaker-specific, perform.

### Continuous (Multimodal) TIMIT speech corpus

The CMT dataset comprises asynchronous, multisession recordings of clean speech from the modalities-air (standard) microphone, bone microphone, and throat microphone. While the air microphone records the speech through lip (and nasal) radiation, the bone microphone is placed in contact with the scalp behind the ear to capture the speech transmitted through bone conduction. The throat microphone is a transducer placed in contact with the skin near the larynx region of the throat. This dataset is custom-built for this study since, to the best knowledge of the authors, no trimodal datasets comprising the air, bone, and throat microphones are available in the public domain. However, bimodal datasets involving air and bone or air and throat speech are available. 30 TIMIT sentences are recorded in multiple sessions from each of the 17 (6 female and 11 male) volunteers in the age group of 18–25 at a sampling rate of 16 kHz and 16 bits/sample. There are about 5600 s of speech data for the three modalities (about 1865 s per modality). A total of 2244 sentences are recorded for the three modalities. For each modality there are 748 utterances (44 utterances/speaker/modality). After performing voice activity detection using frame energy and zero crossing rate to remove non-speech regions, 99555 RP embeddings are extracted for all the three modalities put together. The 33185 RP embeddings of each modality are then split into train:validation:test in the ratio of 70:10:20. Each unimodal system for each of the (Air/Bone/Throat) modality is trained on 23230 RPs, and tested on 6637 RPs. The details of the recording are given in Table 1.

The non-invasive recordings of speech for development of the corpus was carried out as per the relevant guidelines and regulations with the approval of the institutional—Ethical Committee for Scientific Work. Informed consent of the participants was taken prior to recording. The anonymity of the data, the voluntary nature of participation and their right to withdraw from the study was informed to the participants.

**Table 1:** Details of recording CMT datasets.

Air Mic./freq range	HP B4B09M/20 Hz–20 kHz
Throat Mic./freq. range	Sundely Military grade/400–3.4 Hz
Bone Mic/freq. range	Sundely Military grade/400–3.4 Hz
Sound card/software	Realtek stereo/ALSA
Sampling freq./bits per sample	16000 Hz/16
File type	.wav

### Consonant-vowel (unimodal) syllable dataset

The CV dataset^64^ comprises isolated syllabic utterances of 145 consonant-vowel (CV) units of the Indian Language, Hindi, recorded using the air microphone. These CV units can be broadly categorized under 5 vowels /a/, /e/, /i/, /o/ and /u/. With 75 utterances of each CV unit, recorded at 16kHz sampling frequency from 5 speakers (15 utterances for each CV unit per speaker), there are a total of 10875 utterances of the 145 CV units. As mentioned earlier, the purpose of using the CV dataset in this study is that the chaotic behavior of the vocal tract system is more pronounced during the sustained vocalization (associated with the pronunciation of vowels)^65^. We look for speaker characterization during sustained vocalization using different nonlinear parameters, including our RP embeddings $A^L(T)$. Both the datasets in this study are built under laboratory conditions as opposed to unconstrained conditions, as the focus is on representing the nonlinear dynamics of the vocal tract system of a speaker in various models of speech rather than dealing with noisy data. There is no class imbalance in both datasets. A train, validation, and test split of 70:10:20 is used.

### Features used in this study

RP portraits are derived from the raw speech signals by sliding a non-overlapping window of size 37.5 ms (600 samples). This window size has been empirically determined to obtain the RPs that have the best inter-class discrimination. The RP portrait is of size 600 $\times$ 600. The RP portraits are obtained only from the voiced segments, obtained by performing voice activity detection.

#### Choice of embedding dimension and delay parameter for RP

The empirical estimation of the embedding dimension, *n*, of the reconstructed (vocal tract state) vector is given in^66^ that uses the count of the False Nearest Neighbors (FNN). When the dimension *n* is increased, the distance $d_n$ between the pairs of points (*i*, *j*) (state vectors) change. If $\Vert \frac{d_{n}^{(i,j)}}{d_{n-1}^{(i,j)}}\Vert > \epsilon$ (a heuristic threshold) then the points *i* and *j* are called FNN. The desired *n* is that for which $\Vert \frac{d_{n}}{d_{n-1}}\Vert$ yields zero FNN.

The mutual information of the speech samples $s_i$ and $s_{i + \tau }$ (with $\tau$ as the delay parameter) is given by:

11 $$\begin{aligned} I = \sum _{i=1}^{N-\tau } P_{ij} \bigl ( s_{i}, s_{i + \tau } \bigl ) \log _{2} \biggl ( \frac{P_{ij}(s_{i}, s_{i+\tau })}{P_i(s_{i})P_j(s_{i+\tau })}\biggl ) \end{aligned}$$

where $P_{ij}(s_{i}, s_{i + \tau })$ is the joint probability that observation $s_i$, falls into the *i*th interval and the observation $s_{i+\tau }$ falls in the *j*th interval. The first minimum of *I* gives the delay parameter $\tau$^67^. In our study, we use $n=2$ and $\tau =6$ to compute the RPs, $T_{ij}$. RP embeddings learnt by the CNN models, for an input RP portrait, $A^L: f(T)$, contains significant speaker discriminating features as seen in Fig. 3.

These nonlinear RP embedding features are compared with two of the most popular linear spectral features, the spectrogram and Mel Frequency Cepstral Coefficients (MFCC)^68,69^. While spectrograms of size $432 \times 288$ are computed from the CMT dataset with 3 s siding windows, 39 dimensional (13 static, 13 delta and 13 double delta) MFCC are computed using a sliding window of 25 ms with an overlap of 10 ms.

Thus, the RP embeddings, spectrograms and MFCC are the contemporary features used for building the unimodal systems. For the RP embedding based bimodal systems, fusion is carried out at three levels—input level, feature level and score level. For the input fusion, the RP portraits of two modes (air–bone, air–throat and bone–throat) $[T_{air}, T_{bone}], [T_{air}, T_{throat}], [T_{bone}, T_{throat}]$ are fused together to form a two channel input matrix. The feature level fusion is obtained by concatenating the RP embeddings to form three sets of features : $[A^L_{air}, A^L_{bone}], [A^L_{air}, A^L_{throat}]$ and $[A^L_{bone}, A^L_{throat}]$ for the three bimodal systems respectively. The score level fusion is obtained using the sum of normalized scores given by $\underset{s}{\arg \max }\left( \sum _{S}\frac{y^m_S - \mu ^m}{\sigma ^m} \right)$ where *S* is the #speaker, *y* is the individual output score, $\mu ^{m}$ and $\sigma ^{m}$ are the mean and variance for mode *m*. For the trimodal systems built using RP embeddings of the three modes of speech namely (air, throat, bone), apart from the three levels of integration mentioned above for the bimodals, decision level integration is used too.

### Speaker identification experiments

For each of the three modes (air, bone, and throat), the RP portraits of all the speakers are pooled and split into train:validation:test sets in the ratio of 70:10:20. In these experiments, during testing, the different speaker models are tested with a short segment (37.5 ms) of speech (represented by a single RP portrait) of a speaker instead of an entire test utterance. 10-fold cross validation is carried out. Most of the below-mentioned experiments are implemented in PyTorch and some in Keras. A detailed analysis of the results of each of the following experiments is given in “Experimental results” section.

#### Unimodal systems with RP embeddings

To overcome the lack of a huge training dataset that deep neural networks require, we use transfer learning using networks pretrained on the ImageNet dataset^70^ as an alternative to augmenting the dataset with synthetically generated speech. For each of the three modes of speech of the CMT dataset, speaker identification experiments are performed using the pretrained models VGG-16, ResNet-50, Inception-v3, and EfficientNet-B5. RP portraits obtained from the respective modes $T_m$ form the input. These models learn the RP embedding feature space, $A_m^L: f(T_m), A^L \in R^{d \times d}$. The embeddings learned by these models are such that the embeddings of the same class are closer to each other while the embeddings of different classes are farther apart, as reflected by the results in Table 2.

#### Unimodal systems with spectrogram

Here, speaker identification experiments are performed on the spectrograms obtained from the CMT dataset using the VGG-16, ResNet-50, Inception-v3, and EfficientNet-B5 models, for each of the three modes of speech. The trained models are evaluated on spectrograms derived from the 20% test pool. Here the trained models are tested with 3 s of speakers’ data (since each spectrogram image is obtained from 3 s of data), in contrast to the RP unimodal experiments, where the models are tested with shorter segments (37.5 ms) of speakers’ data. Table 3 shows the results of these experiments.

#### Unimodal systems with MFCC

These speaker identification experiments use a Deep Neural Network (DNN) on the 39-dim MFCC features derived from the CMT dataset for each of the three modes of speech. The architecture of the DNN used here with ReLU activation function (R) and dropout layers is 512R-1024R-512R-dropout(0.3)-128R-64R-dropout(0.2)-20S, where S is the final softmax layer^69^. The results are given in Table 4.

#### Bimodal systems with RP embeddings

The bimodal systems use a fusion of two modes either of- air and bone, air and throat, bone and throat. The fusion is implemented at three levels- input level, feature level and score level. This is done to explore the feasibility of using deep learning algorithms (used in the unimodal systems) for combining the information from two different modes of speech which could be complementary, and leverage it to perform speaker recognition tasks.

We build three bimodal speaker recognition systems, one for each pair of modes as mentioned above. For input level fusion, the RP/Spectrogram images of each mode are considered as one channel of the image. The input level fusion causes the final convolutional layer to learn the joint (bimodal) feature space. For feature level fusion, the RP embeddings learnt by two individual streams of convolutional layers (one for each mode *m* are concatenated to form a combined RP embedding $A^L_{{m_1}, {m_2}}: f(T_{m_1}, T_{m_2}) \in \mathbb {R}^{2d}$. This joint RP embedding projects the speaker’s voice characteristics in the $A^L_{m_{1}}$ and $A^L_{m_{2}}$ feature spaces into a joint feature space. The model learns this joint feature space to better capture the speaker-specific voice characteristics to improve the performance of the speaker recognition system.

The score level fusion experiment combines the scores obtained from the speaker recognition models trained on the RP features of two different modes separately. We get three sets of combinations for each of the three bimodal systems. The score level fusion is done as mentioned in “Features used in this study” section Features used in this study. Table 8 gives the comparative performance of the input level, feature level, and score level fusion approaches using the RP embeddings.

#### Bimodal systems with spectrogram

Similar to the RP-based bimodal systems, we build three spectrogram-based bimodal (air and bone, air and throat, bone and throat) systems using input-level, feature-level, and score-level fusion techniques. The input-level fusion is implemented using the (empirically chosen—best performing model from Table 3) Inception-v3 model. For the feature-level fusion, the air, bone, and throat feature streams are implemented using the convolutional layers of ResNet-50, Inception-v3, and Inception-v3, respectively. They have been chosen based on their superior unimodal performance in the respective modes (refer Table 3). The score-level fusion combines the scores from the above-mentioned respective models. The results are given in Table 9 in terms of accuracy.

#### Bimodal systems with MFCC

The 39-dim MFCCs of each of the three modes are combined at the input level, feature level, and score level to create bimodal systems. The DNN architecture used for each fusion is the same as the one mentioned in section “Unimodal systems with MFCC”. For the input-level fusion, a 78-dim MFCC feature is formed by concatenating a pair of 39-dim MFCCs of the corresponding speech features from two modes, thus forming three sets of concatenated feature vectors, one for each pair of modes. The feature-level fusion is formed by passing the pair of 39-dim MFCCs through two separate DNN streams (one for each mode), and then concatenating the flattened outputs. This is used as the input to the dense layers, followed by the final softmax layer for classification. The score level fusion combines the normalized scores of individual unimodals. Table 10 gives the results.

#### Trimodal systems with RP embeddings

These experiments explore the idea that when the nonlinearities of the vocal tract system (captured using RP embeddings) extracted from the complementary air transmitted speech, bone conduction speech, and skin conduction speech are combined, the speaker characteristics would be best captured, compared to unimodal and bimodal systems. The integration is done at four levels—Input level, feature level, score level, and decision level (voting). For the input level integration, the RP portraits of the three modes are stacked to form the input Inception-v3 network used here. For the feature level fusion, while ResNet-50 is used for air mode, Inception-v3 is used for bone and throat modes. These architectures are chosen based on the best performance in the respective unimodal systems. A simple voting mechanism is used for decision-level integration.

#### Trimodal systems with spectrogram

Similar to the RP embeddings, the spectrogram of the three modes are combined at all the four levels mentioned above. The input level fusion uses stacked spectrograms of 432 $\times$ 288 $\times$ 3 pixels as input to the ResNet-50 model. For the feature level fusion, ResNet-50 and Inception-v3 models are used for the convolutional layers of the air, bone, and throat modes, respectively. While score-level fusion is implemented as mentioned in “Choice of embedding dimension and delay parameter for RP” section. Decision level integration is performed with a simple voting mechanism.

#### Trimodal systems with MFCC

Like the above sections, MFCC-based trimodal systems are built using the same four levels of integration. For the input level fusion, the input to the DNN is a concatenation of 39-dim MFCCs of the three modes. The feature-level fusion is obtained using the 3 DNN streams, one for each mode. The flattened output of the CNN layer forms the input to the dense layers, followed by classification by the softmax layer. The score and decision level integration mechanisms are, as mentioned earlier, using the three modes. Table 11 compares the trimodal systems using the RP embeddings, spectrograms, and MFCCs for the four levels of integration.

#### Speaker recognition studies on CV dataset

**Figure 5:**
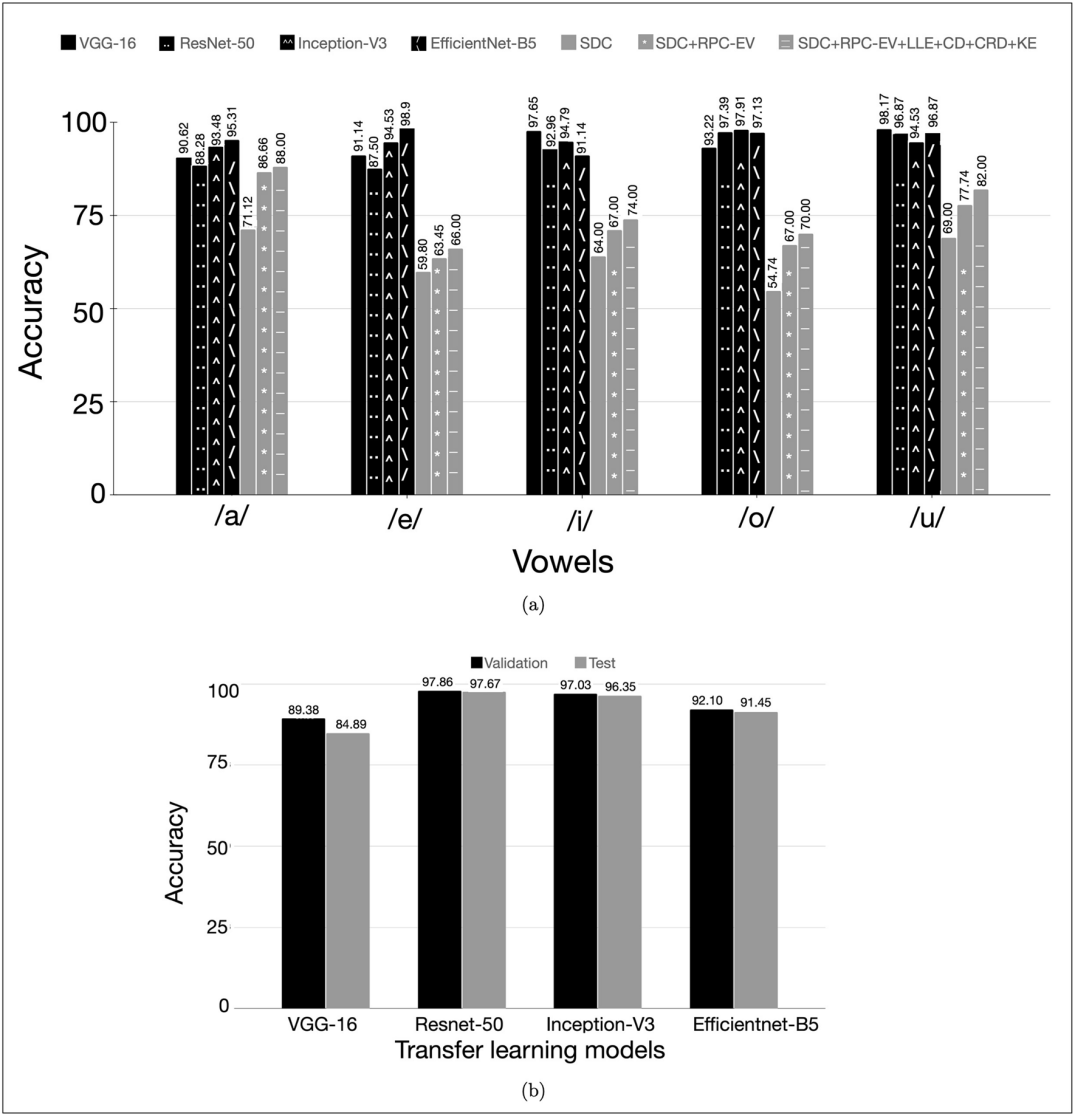
The speaker recognition results using the CV dataset. (**a**) compares the accuracy of the proposed RP embedding as a stand-alone nonlinear feature against other nonlinear features mentioned in literature for the vowel-specific speaker recognition system, and (**b**) shows the good generalization performance of all transfer learning models for the speaker recognition study using the overall (all CV utterances, irrespective of vowels, of each speaker pooled together to form his/her data, as opposed to that mentioned in (**a**)) CV dataset.

The proposed nonlinear RP embeddings feature is compared with other nonlinear features on sustained vocalization. In earlier studies quantification of the nonlinear dynamics using certain measures like Fractal Dimension, Kolmogorov Entropy, Lyapunov Exponents^21,22^, correlation dimension^71^ have been carried out for speaker recognition. We compare the unimodal systems trained on the CV dataset with a similar study in^23^ on the vowel dataset /a/, /e/, /i/, /o/, /u/ which employs these quantification measures combining them with their proposed spectral decay coefficient. The results are shown in Fig. 5 in terms of accuracy.

## Experimental results

As the dataset is a balanced one, accuracy is used as a measure of performance in this study. From Tables 3, 4, 5, 6 and 7, it can be seen that speaker recognition models used in all the unimodal, bimodal, and trimodal experiments can generalize well and that there has been no overfitting. Accuracy is used as a performance metric in this work, since the dataset is class-balanced. Here microprecision.

Table 2 shows the performance of the unimodal speaker recognition systems using the RP embeddings for all three modes of speech. Each of the four models—Inception-v3, ResNet-50, VGG-16, and EfficientNet-B5 generalizes well and, as shown by the similar performances on the validation set and the test set. Inception-v3 gives the best performance for all the three modes, with a test accuracy of 94.43%, 95.58%, and 95.73% for air, bone, and throat modes, respectively. Bone and throat-based unimodal systems have a slightly better accuracy over the air speech, which could be attributed to the position and the placement of the microphones. The excellent performance of the bone and throat speech-based speaker recognition systems show significant speaker-specific information in the speech transmitted via the bone conduction and skin conduction (throat) microphones and that the RP embeddings capture the system nonlinearities better in these modes.

**Table 2:** Unimodal results for CMT corpus using RP.

Model	Air		Bone		Throat	
	Val_Acc%	Test_Acc%	Val_Acc%	Test_Acc%	Val_Acc%	Test_Acc%
VGG-16	93.24 ± 0.25	92.19 ± 0.5	95.34 ± 0.60	94.5 ± 0.32	93.65 ± 0.26	87.97 ± 0.3
ResNet-50	92.51 ± 0.88	93.06 ± 0.74	96.01 ± 0.44	94.87 ± 0.24	91.36 ± 0.27	88.24 ± 0.07
Inception-v3	**96.22** ± **0.12**	**94.43** ± **0.28**	**96.43** ± **0.08**	**95.58** ± **0.04**	**96.04** ± **0.08**	**95.73** ± **0.15**
EfficientNetB5	89.38 ± 1.6	89.32 ± 1.56	91.87 ± 0.83	94.01 ± 0.62	88.75 ± 0.7	90.36 ± 0.92

Highest accuracy values are in [bold].

**Table 3:** Unimodal results for CMT corpus using Spectrogram.

Model	Air		Bone		Throat	
	Val_Acc%	Test_Acc%	Val_Acc%	Test_Acc%	Val_Acc%	Test_Acc%
VGG-16	88.13 ± 1.04	86.41 ± 1.46	97.98 ± 0.44	97.86 ± 0.23	98.75 ± 0.1	99.22 ± 0.07
ResNet-50	**93.75** ± **0.91**	**95.31** ± **0.72**	91.87 ± 0.88	90.10 ± 0.76	98.75 ± 0.1	98.18 ± 0.15
Inception-v3	80.62 ± 1.23	83.59 ± 1.02	**99.37** ± **0.05**	**98.18** ± **0.02**	**99.37** ± **0.01**	**99.74** ± **0.02**
EfficientNetB5	90.62 ± 0.11	92.97 ± 0.34	99.37 ± 0.03	98.18 ± 0.1	98.12 ± 0.16	98.42 ± 0.22

Highest accuracy values are in [bold].

**Table 4:** Unimodal results for CMT corpus using MFCC.

Model	Air		Bone		Throat	
	Val_Acc%	Test_Acc%	Val_Acc%	Test_Acc%	Val_Acc%	Test_Acc%
DNN	91.20 ± 0.04	86.97 ± 0.01	90.12 ± 0	87.48 ± 0.02	98.91 ± 0.03	**96.25** ± **0.05**

Highest accuracy values are in [bold].

Tables 3 and 4 give the performance of the three unimodal systems based on Spectrograms and MFCC, respectively. From Table 3, we see that among the four pretrained models used, Inception-v3 gives the best performance for the bone and throat data, with 98.18% and 99.74% respectively, while ResNet-50 gives the best performance for air data, with 95.31% accuracy. Among the MFCC-based unimodal systems, throat data has the highest test accuracy of 96.25%. From Tables 2, 3, and 4, we can see that the RP embeddings as a nonlinear feature (without combining any linear feature) give similar performance.

Tables 5, 6, and 7 give the performance of the bimodal feature fusion using RP, Spectrogram, and MFCC, respectively. Inception-v3 being the best across unimodal systems, was used for the RP-based bimodal systems. Air–throat performs the best with a test accuracy of 98.21%. In the case of Spectrogram-based systems, ResNet-50 was used for Air, and Inception-v3 was used for Bone and Throat. Air–bone bimodal system performs the best for Spectrogram and MFCC-based fusion, giving a test accuracy of 98.21% and 97.84%, respectively.

**Table 5:** Bimodal results for CMT corpus using RP.

Model	Air–bone		Bone–throat		Air–throat	
	Val_Acc%	Test_Acc%	Val_Acc%	Test_Acc%	Val_Acc%	Test_Acc%
Inception-v3	97.05 ± 0.1	97.35 ± 0.12	97.90 ± 0.05	97.56 ± 0.24	98.14 ± 0.2	**98.21** ± **0.12**

Highest accuracy values are in [bold].

**Table 6:** Bimodal results for CMT corpus using Spectrogram.

Model	Air–bone		Bone–throat		Air–throat	
	Val_Acc%	Test_Acc%	Val_Acc%	Test_Acc%	Val_Acc%	Test_Acc%
ResNet (A), Inception (B,T)	98.00 ± 0.09	**98.21** ± **0.04**	98.52 ± 0.36	96.39 ± 0.52	99.10 ± 0.15	93.01 ± 0.31

Highest accuracy values are in [bold].

**Table 7:** Bimodal results for CMT corpus using MFCC.

Model	Air–Bone		Bone–Throat		Air–Throat	
	Val_Acc%	Test_Acc%	Val_Acc%	Test_Acc%	Val_Acc%	Test_Acc%
DNN	97.98 ± 0.16	**97.84** ± **0.08**	97.08 ± 0.04	96.40 ± 0.1	97.04 ± 0.13	97.12 ± 0.17

Highest accuracy values are in [bold].

**Table 8:** Bimodal results for different fusions for RP.

Fusion mechanism	RP		
	Air–Bone	Bone–Throat	Air–Throat
Input-Fusion	99.29 ± 0.02	95.63 ± 0	**99.61** ± **0.04**
Score-Fusion	97.82 ± 0.2	98.36 ± 0.26	**99.07** ± **0.17**
Feature-Fusion	97.35 ± 0.12	97.56 ± 0.24	**98.21** ± **0.12**

Highest accuracy values are in [bold].

**Table 9:** Bimodal results for different fusions for Spectrogram.

Fusion mechanism	Spectrogram		
	Air–Bone	Bone–Throat	Air–Throat
Input-Fusion	96.29 ± 0.74	**98.84** ± **0.07**	97.91 ± 0.14
Score-Fusion	97.81 ± 0.22	98.36 ± 0.41	**99.06** ± **0.16**
Feature-Fusion	**98.21** ± **0.04**	96.39 ± 0.52	93.01 ± 0.31

Highest accuracy values are in [bold].

Tables 8, 9, and 10 show the results of the bimodal systems built using the three mechanisms of integration at the input level, feature level, and score level for RP, spectrogram, and MFCC, respectively. The performance of the join RP embedding system can be compared with that of the spectrogram and MFCC. For the joint RP embeddings, among the three bimodal at each level of integration, the bimodal (air–bone and air–throat) systems involving the air speech give the best performances (99.2% and 99.6% respectively) when integrated at the input level, the third bimodal system using only the alternate speech (bone–throat) gives the best performance (98.36%) for score level fusion. The MFCC feature-based bimodal system gives very good results (99.28% and 100% for bimodal systems, respectively) using the input level and score level fusion. In contrast, spectrogram based bimodal gives 98.84% and 99.06%, respectively, for the bone–throat and the air–throat bimodal systems using the input level and score level fusion. The relatively marginal drop in the performance of the spectrogram-based systems could be attributed to the relatively smaller dataset of spectrograms (one spectrogram corresponds to 3 *s* of speech data). The good generalization ability of the feature-fusion-based bimodal systems built using RP, spectrogram, and MFCC are illustrated in Tables 5, 6, and 7, respectively. The proximity between the validation and test accuracy can be observed in each case.

The performance of the trimodal systems for all the three features - the proposed RP embeddings, the spectrograms, and MFCC is seen in Table 11 for four levels of integration - input level, feature level, score level, and decision level (voting). While the best performing trimodal for RP embeddings is 99.84% using input fusion, it is 99.45% and 100% using score fusion for spectrograms and MFCC, respectively. The joint feature space of the RP embeddings is more speaker-specific than the individual RP embedding space (99.84% vs. 95.58% respectively). A similar trend is observed with the other two features(spectrogram and MFCC). Note that in all the bimodal and trimodal experiments, the best performing unimodals for the respective modes have been used.

**Table 10:** Bimodal results for different fusions for MFCC.

Fusion mechanism	MFCC		
	Air–bone	Bone–throat	Air–throat
Input-Fusion	**99.28** ± **0.03**	97.12 ± 0.12	97.84 ± 0.02
Score-Fusion	99.28 ± 0.04	**100** ± **0.00**	99.28 ± 0.2
Feature-Fusion	**97.84** ± **0.08**	96.40 ± 0.1	97.12 ± 0.17

Highest accuracy values are in [bold].

**Table 11:** Trimodal results for different fusions for RP, Spectrogram and MFCC.

Fusion mechanism	Recurrence plot	Spectrogram	MFCC
	Test_Acc	Test_Acc	Test_Acc
Input-fusion	**99.84** ± **0.03**	92.59 ± 0.04	97.84 ± 0.1
Score-fusion	98.45 ± 0	**99.45** ± **0.02**	**100** ± 0.00
Feature-fusion	99.53 ± 0.14	98.75 ± 0.02	99.28 ± 0.06
Decision-fusion	95.87 ± 0.17	95.87 ± 0.11	98.73 ± 0.08

Highest accuracy values are in [bold].

The comparative prediction times of the unimodal, bimodal and trimodal systems using RP embeddings, Spectrogram and MFCC, respectively, are given in Table 12. Each is an average prediction time obtained by predicting 10 test samples. From the table, it is seen that the prediction time increases from unimodal to bimodal to trimodal, across all three features. In all three cases, unimodal, bimodal and trimodal, the RP embeddings and Spectrogram based systems have similar testing times, and are slower by a factor of up to 30% compared to the faster MFCC based systems. This is attributed to the loading time of individual images during test time, in the former two cases, compared to MFCC features. However, even the slowest prediction time, of RP embedding bimodal and trimodal system is still 0.5 seconds, which makes it feasible to operate in real time. The experiments were run on K80 GPU on Google Colab.

**Figure 6:**
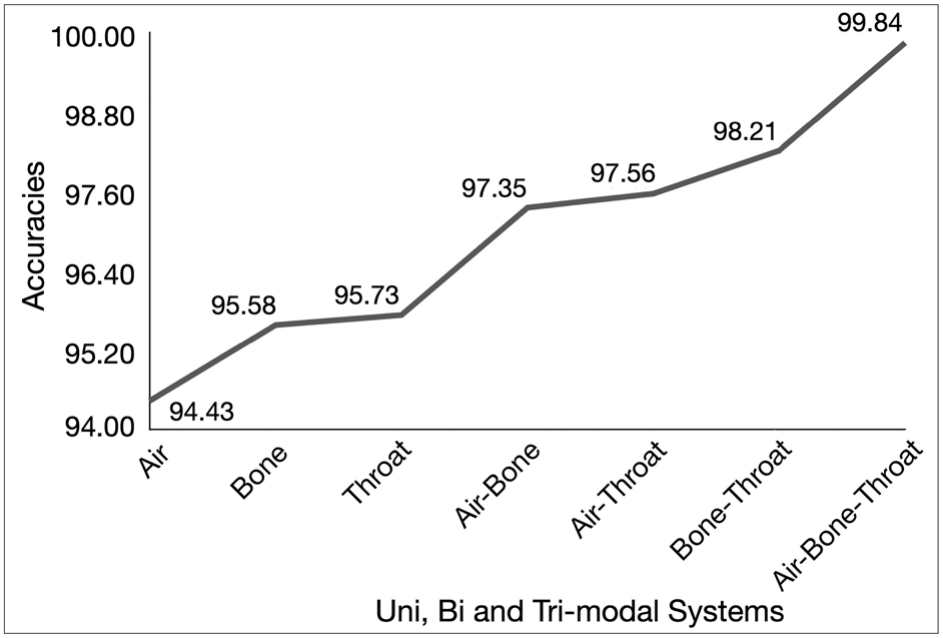
Performance gain of RP based bimodal and trimodal systems over unimodal systems.

From the experiments so far (Tables 2, 3, 4, 5, 6, 7, 8, 9, 10, 11), the evidence for RP embeddings being a standalone, nonlinear feature for speaker modeling is strong, and on par with the linear features (like MFCC). The improvement in performance in standalone RP based systems as the modes are combined, for unimodal, bimodal and trimodal systems is depicted in Fig. 6. To study the possible presence of complementary, speaker-discriminating characteristics in the nonlinear and linear features, RP embeddings are combined with popular linear features, MFCC and Gamma Tone Frequency Cepstral Coefficients (GFCC) to build the RP-MFCC and RP-GFCC unimodal, bimodal, and trimodal speaker recognition systems. A review of, earlier work on speaker recognition that combined nonlinear and linear features showed that, while linear features outperformed nonlinear features by a large margin, their combination provided a small improvement in performance over that of linear features. The nonlinear feature contributed only marginally to the already outperforming linear feature based systems. In contrast, in this work, the equally well performing nonlinear and linear features (as seen in the first three rows of Table 13) are combined, as mentioned above. The RP embeddings and the MFCC/GFCC embeddings, obtained from independent CNNs, are concatenated to form the (RP-MFCC and RP-GFCC) joint features for speaker classification. The joint feature space yields results similar to that of RP embeddings, at best. While the RP-MFCC joint feature shows no improvement over the RP embeddings, the RP-GFCC feature shows a relative dip in performance, refer Tables 13 and 14. This lack of significant improvement in performance in the combined features could be due to the absence of complementary evidence in the RP embeddings and the MFCC/GFCC embeddings, though they have a commendable individual performance. However, when the linear features MFCC and GFCC are combined, the MFCC–GFCC systems show a marginal improvement over RP systems by up to 0.8% for unimodal (bone, throat) systems and for two of the bimodal systems, although for the trimodal system RP outperforms MFCC–GFCC by 1.2% (Tables 13, 14).

**Table 12:** Prediction time computation.

Recurrence plots		Spectrogram		MFCC	
Unimodal		Unimodal		Unimodal	
Air	0.18	Air	0.13	Air	0.05
Bone	0.26	Bone	0.19	Bone	0.04
Throat	0.19	Throat	0.17	Throat	0.04

Bimodal		Bimodal		Bimodal	
Air–bone	0.38	Air–bone	0.28	Air–bone	0.05
Bone–throat	0.42	Bone–throat	0.34	Bone–throat	0.05
Air–throat	0.36	Air–throat	0.29	Air–throat	0.05

Trimodal		Trimodal		Trimodal	
Air–bone–throat	0.56	Air–bone–throat	0.43	Air–bone–throat	0.06

**Table 13:** Performance comparison of RP + MFCC and RP + GFCC unimodal systems over stand-alone nonlinear (RP) and linear (MFCC and GFCC) systems. Note that stand-alone systems over MFCC repeated here for comparison.

Feature	Air		Bone		Throat	
	Val_Acc%	Test_Acc%	Val_Acc%	Test_Acc%	Val_Acc%	Test_Acc%
RP	96.22 ± 0.12	**94.43** ± **0.28**	96.43 ± 0.08	95.58 ± 0.04	96.04 ± 0.08	95.73 ± 0.15
MFCC	91.20 ± 0.04	86.97 ± 0.01	90.12 ± 0	87.48 ± 0.02	98.91 ± 0.03	96.25 ± 0.05
GFCC	81.67 ± 1.5	84.85 ± 1.2	65.693 ± 1.2	80.29 ± 1.8	82.29 ± 0.42	85.08 ± 0.48
MFCC + RP	96.22 ± 0.16	94.43 ± 0.20	96.43 ± 0.08	95.58 ± 0.14	96.04 ± 0.10	95.73 ± 0.06
GFCC + RP	86.71 ± 0.31	82.37 ± 0.53	91.08 ± 0.14	89.04 ± 0.58	85.94 ± 0.72	82.25 ± 0.48
MFCC + GFCC	92.969 ± 0.08	94.07 ± 0.12	96.10 ± 0.04	**96.18** ± **0.04**	97.26 ± 0.1	**96.50** ± **0.2**

Highest accuracy values are in [bold].

**Table 14:** Performance comparison of RP + MFCC and RP + GFCC bimodal systems over stand-alone nonlinear (RP) and linear (MFCC and GFCC) systems. Note that stand-alone systems over MFCC repeated here for comparison.

Feature	Air–bone		Bone–throat		Air–throat		Air–bone–throat	
	Val_Acc%	Test_Acc%	Val_Acc%	Test_Acc%	Val_Acc%	Test_Acc%	Val_Acc%	Test_Acc%
RP	97.05 ± 0.19	97.35 ± 0.07	97.90 ± 0.22	97.56 ± 0.1	98.14 ± 0.38	98.21 ± 0.14	99.92 ± 0.2	**99.84** ± **0.03**
MFCC + RP	97.05 ± 0.02	97.35 ± 0.01	97.91 ± 0.04	97.56 ± 0.24	98.14 ± 0.33	**98.21** ± **0.47**	99.38± 0.14	99.53 ± 0.05
GFCC + RP	92.56 ± 0.32	91.77 ± 0.87	93.45 ± 0.61	93.54 ± 0.16	89.31 ± 0.72	88.78 ± 0.52	93.66 ± 0.19	93.97 ± 0.38
MFCC + GFCC	97.89 ± 0.30	**97.47** ± **0.27**	99.09 ± 0.01	**98.44** ± **0.06**	94.76 ± 0.22	96.11 ± 0.16	98.43 ± 0.11	98.57 ± 0.04

Significant values are in [bold].

Figure 5 shows the results obtained using RP embedding feature on the CV(air speech) dataset. Figure 5a shows the performance of RP embeddings in comparison with the other nonlinear features as mentioned in ﻿section "Speaker recognition studies on CV dataset" for each of the 5 vowels of the Indian language, Hindi. For this experiment, the CV dataset is categorized based on the 5 vowels /a/, /e/, /i/, /o/ and /u/ and speaker recognition studies are performed using each of them.

It can be seen that RP embeddings outperform all the nonlinear features under all the vowel categories. Among the four pretrained models used in this study, VGG-16 gives the highest accuracy of 97.7% and 98.2% on the close front and back vowels /i/ and /u/, respectively. While the highest accuracy for the close-mid front vowels /e/ and central open vowel /a/ (99% and 95.3% respectively) are given by EfficientNet-B5, ResNet-50 gives 97.9% for close-mid back vowel /o/. In contrast, the other nonlinear features when combined perform well with 88% accuracy for vowel /a/ and for the remaining front and back vowels the accuracies are between 50% - 70%. Figure 5b shows the results for the speaker recognition experiment performed on the entire CV dataset (without any splitting based on vowels). The results are similar to the vowel based categorization experiment.

In order to validate the scalability of RP based embeddings for speaker recognition on larger datasets, a standalone study was conducted on the publicly available ABCS corpus comprising synchronously recorded air and bone microphone speech from 100 (50 male and 50 female) speakers^72^. Random, 30 clean speech utterances from each of the 100 speakers for both air and bone conduction were chosen as the dataset. Unimodal experiments similar to those in Tables 2 and 3 and bimodal experiments similar to those in Tables 5-7 are conducted. This results in test accuracies of 90.12% and 90.74% for air and bone conducting microphones, respectively using RPs on an Inception-v3 network. For the bimodal air–bone system the test accuracy is 95.47%. It shows that RPs perform on par with other linear features for larger datasets as well, and that RPs, as speaker discriminating features, are scalable. A more exhaustive study on all the three modalities is desirable though.

## Discussion

Though the CMT dataset used in this work is limited by the size (17 speakers), it covers the necessary variabilities that are speaker-specific and channel variabilities by recording with different channels across multiple sessions. The study has shown the feasibility of learning a nonlinear representation that is speaker-specific across various modes of speech - air conduction, bone conduction, and skin conduction. Given the RP as an input, the convolutional layers of the network learn a (nonlinear) feature space (RP embeddings) that efficiently models the speakers. The study has also shown that learning a joint (either a bimodal or trimodal) representation of the complementary nonlinear cues of two/three modes of speech at various levels of integration yields a better recognition rate.

The RP embeddings both in individual (air/bone/throat ) feature space and the joint feature space have shown significant speaker-specific characteristics on par with the popular linear representations of speakers, namely spectrogram and MFCC. Among the nonlinear speaker-specific features, the superior class-discriminatory capability of the proposed RP embeddings over other nonlinear features that are used in literature for speaker recognition such as Lyapunov exponents, Kolmogorov entropy and correlation dimension measure the nonlinearity in a signal^71,73,74^. While Lyapunov exponents associated with a trajectory measure the mean rates of convergence and divergence of nearby trajectories, correlation dimension quantifies the number of degrees of freedom and the extent of self-similarity in the attractor‘s structure. Kolmogorov entropy defined over a state-space, measures the rate of information loss or gain over the trajectory. These measures search for a signature of chaos in the observed time series (speech, here). Since these measures quantify the structure of the underlying nonlinear dynamical vocal tract system, they are considered as nonlinear features. However, a combination of such nonlinear features for discriminating speakers does not yield the desired results. Even the best performing combined nonlinear feature based model gives an accuracy of only 82%. In contrast, the recurrence plot embeddings give an accuracy of about 98%. A crucial advantage of recurrence plot-based approaches over other nonlinear approaches is that they perform reasonably well even when the length of the time series is short^75^. Moreover, in cases when the underlying system is not sufficiently deterministic or stationary, recurrence plots have proven useful in characterizing their behavior.

The trained speaker models have been tested on single RP embeddings (corresponding to RP portraits derived from 37.5 ms speech segments) of each speaker, as opposed to being tested on entire utterances. The good performance of the unimodal, bimodal, and trimodal systems using the RP embeddings shows that these features are effective for short segment estimates of the speaker models in all the three modes of speech considered in this study. In other words, a 37.5 ms duration of speech is sufficient to identify its speaker with an accuracy $> 99\%$ using the RP embeddings as features.

The joint space of RP embeddings well captures the complementary evidence from the three modes, as seen in the multimodal results. An interesting aspect is the bone–throat bimodal system. This bimodal system learns the joint feature space of the RP embeddings of entirely alternate speech and is similar to the air speech-based unimodal system. We get the best accuracy (among various integration techniques) of 98.36%. Comparatively, bimodal systems that use air speech as one of the modes (air–bone and air–throat bimodal systems) give the best accuracy of 99.6%. This gives credence to the claim that a speaker recognition system can be built and deployed using a combination of entirely alternate speech and joint RP embeddings which performs equivalent to the standard system (using air speech) for short speech segments.

Overall,this work has demonstrated the efficacy of the RP embeddings (for unimodal systems using various speech modalities) and joint RP embeddings (for bimodal and trimodal systems) as stand-alone, nonlinear, short duration features that capture speaker discriminatory features. This work also shows that RP embeddings contain better speaker-specific information than the other nonlinear features used for speech systems.

As a future work, larger multimodal (trimodal) datasets need to be built in (1) the control environment, (2) VoxCeleb (speaker in the wild) type dataset, that captures large and multiple variations, (3) adverse (noisy) environment to build multimodal systems that are robust against the background noise.

## Conclusion

In this paper, a new nonlinear dynamical feature is proposed for speaker modeling. Known as the recurrent plot embedding derived from deep convolutional layers, this acts as a standalone feature, without the necessity for combining linear features to it. To the best knowledge of the authors, RP embedding is the first standalone nonlinear feature to perform on-par with the popular linear features for speaker recognition. The proposed features may be considered as alternative nonlinear dynamic features rather than as a replacement to the standard linear features like MFCC.

Learning joint RP embeddings from 3 different modalities of speech, the air, the bone and the throat conduction, this paper proposed multimodal (bimodal and trimodal) speaker recognition systems that give results comparable to state-of-the-art systems that are built using linear features. The compelling results achieved using RP embeddings from a mere 37.5 ms duration speech utterances suggests that RP embeddings are well suited for short segment speaker modeling, applicable in situations where very less speaker data is available.

In favorable results obtained by the bimodal systems, build using joint RP embeddings of bone and throat signals validate the feasibility of building speech systems in the absence of standard (air conduction) speech. Furthermore, the worst case prediction time is still half a second, making it suitable for real time applications.

## Data Availability

Data sets generated and used in the current study are available from the corresponding author on reasonable request.
